# Food allergy competencies of dietitians in the United Kingdom, Australia and United States of America

**DOI:** 10.1186/2045-7022-4-37

**Published:** 2014-11-14

**Authors:** Kate Maslin, Rosan Meyer, Liane Reeves, Heather Mackenzie, Anne Swain, Wendy Stuart-Smith, Rob Loblay, Marion Groetch, Carina Venter

**Affiliations:** School of Health Sciences & Social Work, University of Portsmouth, Portsmouth, UK; David Hide Asthma and Allergy Research Centre, Isle of Wight, UK; Department of Gastroenterology, Great Ormond Street Hospital for Sick Children, London, UK; Oxford Health NHS Foundation Trust, Oxford, UK; The Allergy Unit at Royal Prince Alfred Hospital, Sydney, Australia; University of Sydney, Sydney, Australia; Icahn School of Medicine at Mount Sinai, New York, USA

**Keywords:** Competency, Dietitian, Food allergy, Knowledge

## Abstract

**Background:**

A knowledgeable and competent dietitian is an integral part of the food allergy multidisciplinary team, contributing to effective diagnosis and management of food allergic disorders. Little is currently known about the food allergy training needs and preferences of dietitians. The purpose of this paper is to measure and compare self-reported food allergy competencies of dietitians based in the UK, Australia and USA.

**Methods:**

A survey of USA-based paediatric dietitians was developed to measure self-reported proficiency and educational needs in the area of food allergy. The survey was modified slightly and circulated online to paediatric and adult dietitians in the UK and Australia. Descriptive statistics and Pearson correlations are presented.

**Results:**

A total of 797 dietitians completed the questionnaire. Competency in “developing food challenge protocols” and “managing feeding problems” were rated the poorest overall across all three settings. A higher level of competency was significantly positively associated with length of practice as a dietitian, percentage of caseload composed of patients with food allergy and training in food allergy. The most popular topics for further training were food additives, pharmacological reactions and oral allergy syndrome.

**Conclusions:**

There is a need amongst dietitians to increase their knowledge in different aspects of food allergy diagnosis and management, specifically the areas of developing food challenge protocols and management of feeding problems. This study provides valuable information for designing targeted food allergy education for dietitians.

**Electronic supplementary material:**

The online version of this article (doi:10.1186/2045-7022-4-37) contains supplementary material, which is available to authorized users.

## Introduction

The main aim in the management of Food Hypersensitivity (FHS) is to prevent the occurrence of acute and chronic symptoms by avoiding the offending food(s), whilst providing a nutritionally balanced diet [1]. In order to ensure effective management of any type of food allergic disorder, an appropriate dietary assessment and avoidance strategy is required [2]. A knowledgeable and competent food allergy dietitian is uniquely qualified to deliver this [3]. In recent years, five official international guidelines have been published on the diagnosis and management of food allergies; the World Allergy Organisation (WAO) guidelines on the diagnosis and management of cow’s milk allergy [4], the USA National Institute of Allergy and Infectious Disease (NIAID) guidelines on the diagnosis and management of food allergies in adults and children [5], the UK National Institute of Health and Clinical Excellence (NICE) guidelines on the diagnosis of food allergies in children [6], the European Society for Paediatric Gastroenterology, Hepatology and Nutrition guidelines on Cow’s Milk Protein Allergy [7] and the Irish Food Allergy Network (IFAN) Paediatric Food allergy guidelines [8]. Although each of these guidelines identifies the importance of a nutrition consultation, only the UK NICE, ESGPHAN and IFAN guidelines recognise that dietitians play a key role in both the diagnosis and management of food allergies.

In practice, the role of the dietitian working in the area of food allergy involves a range of responsibilities, consisting of, but not limited to [9]; taking an allergy-focused diet history and interpretation of skin prick tests, advising on formula choice and complementary feeding including nutrient supplements, allergen avoidance advice including practical advice on substitutes and recipes and monitoring nutritional status. Crucially, the dietitian has a lead role in the planning and design of food challenges for both diagnosis and determination of tolerance. A double blind placebo controlled food challenge remains the gold standard for diagnosis of food allergy [10]. Although in clinical practice, food challenges are typically not double blinded, expertise is required to calculate and translate appropriate doses to acceptable portion sizes. However, a previous survey of dietitians in the USA [11] indicated that despite good knowledge levels in some aspects of food allergy, a significant number of dietitians had no proficiency in developing food challenge protocols. This paper will compare self-reported food allergy competencies of dietitians based in the UK, Australia and USA, by combining previously published data from US –based paediatric dietitians [11] with new data which surveyed both adult and paediatric dietitians based in Australia and the UK.

## Methods

The original survey of USA-based paediatric dietitians undertaken by Groetch *et al*. [11] was developed by a group of expert health professionals from the Consortium of Food Allergy Research (CoFAR), to measure self-reported proficiency and educational needs and preferences of paediatric dietitians. It was piloted, then distributed online to the Paediatric Nutrition Practice Group of the Academy of Nutrition & Dietetics. Respondents were asked to rate their knowledge and competency on a four point scale (high, moderate, low and not at all proficient). Permission to use this data as a published resource, in combination with newly collected data, was granted.

For both the UK and Australia, the questionnaire was modified to address local conditions. A five-point scale was used (high, moderate, low, not at all proficient and N/A in my practice). The questionnaire used in the UK is shown in Additional file 1: Table S1. The questionnaire used in Australia differed slightly as it had separate questions about Food Allergy (FA) and Food Intolerance (FI), where the term ‘food allergy’ was solely used for describing IgE mediated food allergy.

### Sample

The distribution of the survey differed between countries. In the UK, a weblink was posted on the British Dietetic Association’s (BDA) website, which has approximately 7000 members. The questionnaire was also published once in the BDA magazine and emailed once to dietitians who are members of specialist groups.

In Australia the questionnaire was circulated once via a weekly newsletter to all Dietetic Association of Australia (DAA) members, which has approximately 5000 members. A reminder email was sent three weeks later to the Food Allergy and Intolerance, Gastroenterology and Paediatric and Maternal Health Interest groups.

In the UK, the University of Portsmouth ethics committee was consulted, who advised that specific ethical permission was not required to undertake an online survey. In Australia, ethical approval was obtained from the Research Development Office of the Royal Prince Alfred Hospital, New South Wales.

Descriptive statistics are presented. Percentage responses are calculated per question based on the number of respondents answering the question. All statistical analyses were conducted using SPSS version 20.0 (SPSS, Inc., Chicago, ILL, 2012). One-tailed Pearson correlations were calculated to determine if any factors were associated with higher levels of competency.

## Results

### Participant characteristics

A total of 797 dietitians completed the questionnaire. Demographic characteristics of all participants are shown in Table 1.

**Table 1:** **Demographic characteristics of all participants**

Characteristic	Options	UK	Australia	USA
		(n = 336)	(n = 150)	(n = 311) [ [11]]
		%	%	%
Years in practice	0-5 years	31.7	42.0	20.6
	6-10 years	21.7	18.7	14.8
	11-15 years	15.5	12.0	15.1
	>15 years	31.8	27.3	49.5
Practice settings	Hospital (outpatient)	39.0	42.0	46.0
	Hospital (inpatient)*	NA*	40.0	37.6
	Private practice	2.7	32.0	13.2
	Community	36.0	34.0	-
	Industry	0.0	2.0	-
	Food Service	0.0	4.6	-
	Academic	0.3	2.6	-
	Research	0.9	5.3	-
	Other	21.1	4.6	28.3
Caseload composed of food allergy patients**	<10%	31.0**	66.0	57.6
	>10%	69.0**	34.0	42.4
Allergy training	During dietetic training	58.3	77.0	31.0
	Post registration course	17.0	28.0	51.9
	Postgraduate course	5.1	3.0	NA***
CPD resources currently used****	Academic journals	-	89.0	85.1
	Academic websites	-	52.7	59.3
	Dietetic/advocacy groups	-	70.1	72.0
	Conferences	-	70.0	56.0

NA = Not Applicable.

*UK questionnaire did not specify inpatient or outpatient.

**The UK respondents were not directly asked the proportion of their caseload comprised of FA patients. These figures relate to respondents who answered “*not at all*” *or* “*slightly relevant*” *to the question* “*How relevant*/*applicable to your practice were the questions in this survey*?”.

***USA questionnaire did not list “*postgraduate course*” *as an option*.

****UK questionnaire did not ask what CPD resources currently used.

A considerable number of participants worked in an outpatient setting (39%, 42% and 46% of UK, Australia and USA-based dietitians respectively). The majority of dietitians based in UK (58.3%) and Australia (77%) learnt about FA during their basic dietetic training. However in the USA, the majority of respondents (51.7%) learnt about allergy after qualifying as a dietitian.

The results of the UK and Australia questionnaires are compared with the results previously published by Groetch *et al*., [11] in Table 2.

**Table 2:** **Comparison of food allergy knowledge and competencies of dietitians based in the UK**, **Australia and USA**

	High			Moderate			Low			Not at all		
	Aus	USA	UK	Aus	USA	UK	Aus	USA	UK	Aus	USA	UK
Understand FA	25	57	23	45	41	53	18	2	17	8	0	5
Understand FI	43	59	22	45	39	54	10	2	18	1	0	4
Understand diagnosis of FA/FI	18	19	23	41	53	41	30	24	26	8	4	6
Recognise signs and symptoms of FA/FI	25	29	23	50	58	48	20	12	22	3	2	4
Develop Food Challenge protocols	13	8	8	35	35	25	35	38	32	13	19	23
Educate patients on avoidance	30	42	33	39	46	42	25	12	18	3	1	4
Develop elimination diet	18	14	21	23	40	25	22	31	12	18	15	10
Manage Multiple FA	17	28	21	17	49	23	26	20	13	19	3	12
Manage Feeding problems	13	17	9	19	39	25	26	33	19	23	10	13

### Food Allergy topics with high level of competency

**Figure 1:**
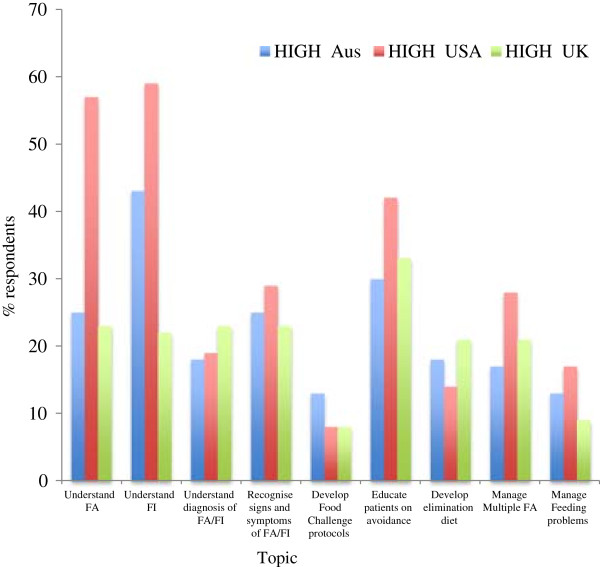
**Food Allergy topics rated with “high” level of competency.**

Topics that were rated as “high” levels of competency are displayed in Figure 1. The USA-based dietitians had the greatest proportion of respondents rating themselves as highly competent for 6 areas (understanding definitions of FA and FI, recognising signs and symptoms, educating patients on avoidance, managing multiple food allergies and managing feeding problems). UK-based dietitians had the greatest proportion of respondents rating themselves highly in 2 areas (understanding diagnosis of FA & FI and developing an elimination diet). Australia-based dietitians had the greatest proportion of respondents rating themselves highly for one area (developing food challenge protocols), however this was only 13% of respondents.

### Food Allergy topics with low levels of competency

The competencies that were rated the poorest overall across all three countries were developing food challenge protocols and managing feeding problems, with 19% and 13% of all respondents respectively rating themselves as “not at all proficient”.

However Pearson correlations calculated for the UK and Australia data indicate that higher competency in the areas of food challenge and managing feeding problems were significantly positively associated with length of practice as a dietitian, percentage of caseload composed of food allergy patients and training in food allergy. The strongest correlation existed between higher competency in managing feeding problems and% of caseload composed of allergy patients (r = 0.50, *p* < 0.01 in UK and r = 0.517, *p* < 0.01 in Australia). There was no correlation between competency in these two areas and setting of workplace. Correlation coefficients are displayed in Table 3.

**Table 3:** **Correlation between competency in food challenge protocols and feeding problems and participant characteristics**

	UK		Australia	
	(n = 336)		(n = 150)	
	Food challenge protocols	Feeding problems	Food challenge protocols	Feeding problems
Years of practice	r = 0.12	r = 0.246	r = 0.204	r = 0.26
	p < 0.05	p < 0.01	p < 0.01	p < 0.01
Setting of practice	r = 0.08	r = 0.02	r = 0.019	r = 0.04
	p = 0.71	p = 0.32	p = 0.40	p = 0.29
Caseload of allergy patients	r = 0.32	**r** = **0.50**	**r** = **0.487**	**r** = **0.517**
	p < 0.01	**p** < **0.01**	**p** < **0.01**	**p** < **0.01**
Specialist allergy conference	**r** = **0.423**	**r** = **0.478**	r = 0.256	r = 0.339
	**p** < **0.01**	**p** < **0.01**	p < 0.01	p < 0.01
FA education/ workshop	r = 0.264	**r** = **0.413**	**r** = **0.416**	r = 0.382
	p < 0.01	**p** < **0.01**	**p** < **0.01**	p < 0.01

Strong positive correlations (r > 0.4) are in bold.

### Further training needed

Respondents in the UK and Australia were asked which specific FA topics they would like further training in. Results are shown in Table 4. Of note, the most popular topics were: reactions to food additives (67% and 73% in the UK and Australia respectively), pharmacological reactions (66% and 70% in the UK and Australia respectively) and oral allergy syndrome (62% and 68% in the UK and Australia respectively).

**Table 4:** **Food allergy and intolerance training needs of UK and Australia**-**based dietitians**

Topic	UK (%)	Australia (%)
	n = 336	n = 150
Reactions to food additives	67	73
Pharmacological reactions (e.g. salicylates)	66	70
Oral Allergy Syndrome	62	68
Management of Irritable Bowel Syndrome	53	48
Cereal allergy	47	52
Cows' milk protein allergy	39	50
Soy allergy	46	39
Nut and seed allergy	45	42
Fish/shellfish allergy	43	38
Egg allergy	37	38
Coeliac disease	21	29
Lactose intolerance	34	14

### Educational resources needed

**Figure 2:**
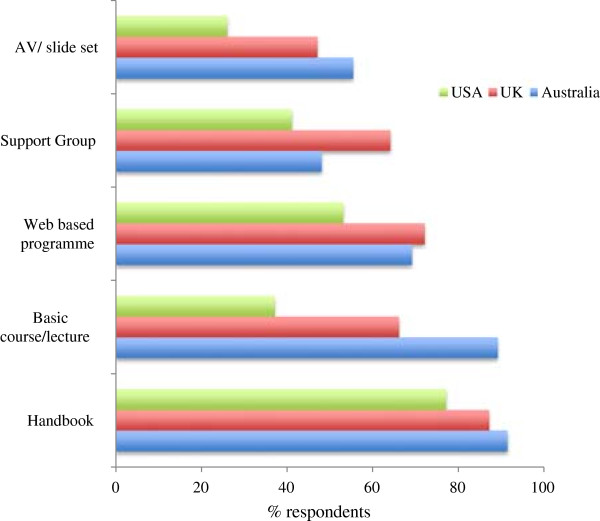
**Preferred Food Allergy educational resources for dietitians.**

When asked what resources they would be “very likely” or “likely” to use to improve their knowledge of FA; a handbook, basic course and web-based programme were the most popular choices. Results are displayed in Figure 2.

## Discussion

This study set out to compare self-reported food allergy knowledge and competencies of dietitians in the UK, USA and Australia, by combining previously published data from USA –based paediatric dietitians [11] with new data from Australia and the UK. Overall we found evidence of suboptimal levels of knowledge and competency in several key food allergy aspects across all three countries.

The original questionnaire used by Groetch *et al*. [11] was developed to identify the self-reported food allergy proficiency and education needs of paediatric dietitians in the United States. Similarly, the later two questionnaires were administered to both adult and paediatric dietitians in Australia and the UK to establish a baseline of knowledge and competencies in order to advance the education and training of dietitians in the area of food allergy. This is in acknowledgement of the pivotal role dietitians play in the diagnosis and management of both adults and children with food allergy.

Although the questionnaires were made available to dietitians working in all clinical specialities and those not working in food allergy were encouraged to respond, only 5% of the UK-based respondents reported the questionnaire was ‘not at all relevant’ to their practice, indicating that knowledge of food allergy is broadly relevant to the vast majority of UK-based dietitians, even if they are working in another clinical speciality. More than 50% of the Australia-based respondents were working with paediatric or adult food allergy patients at the time of the survey, again emphasising how food allergy pervades across dietetic practice. Similarly, 90% of the USA-based sample worked with food allergy patients, however this could be skewed by the fact that only paediatric dietitians were recruited in the USA and it is well known that food allergy is more prevalent in children that adults [12].

The differences seen between countries could be explained by differences in dietetic training internationally. A greater percentage of Australia and UK based dietitians than USA based dietitians, reported to have learnt about FA during basic dietetic training. Attempts have been made to standardise the undergraduate and postgraduate training of nutrition and dietetic professionals across the world [13, 14]. However, a report from The International Confederation of Dietetic Associations (2008) [14] highlighted the heterogeneity of dietetic training and practice in different countries in terms of level of basic education, practical experience, competency standards and scope of practice. The importance of establishing internationalism in dietetic training in order to produce practitioners that are competent to manage emerging diseases has previously been raised [15].

A key trend emerging from these three questionnaires is the discrepancy in knowledge across different aspects of FA diagnosis and management. The public confusion that exists between perceived and actual food allergy may be contributing to this problem [16]. Although some aspects of FA management (e.g. educating patients about food avoidance, recognising signs and symptoms, understanding definitions) were well rated, others such as developing food challenges were rated poorly across all three cohorts. This was particularly the case in the UK-based cohort, where half of respondents who reported that the questionnaire was “moderately or very” relevant to their practice, rated their competency level to be “low” or “not at all proficient”. This is extremely critical to the progression of allergy services in the UK, in order to ensure that patients are correctly diagnosed and timely monitored for determining tolerance to food allergens [17]. Without the availability of trained health professionals to design and implement food challenges, it is likely that patients may be incorrectly diagnosed and placed on an exclusion diet unnecessarily. Indeed a lack of allergy services providing appropriately designed hospital-based food challenges may mean that unsafe home reintroduction challenges will be advocated, thus putting patients at risk. Reassuringly, there was a strong positive correlation between attendence at a specialist FA conference or education/workshop and competency in the area of food challenges.

Our findings are in agreement with research that has been conducted in other health professional groups across the world. A study of doctors (n = 1317) in the UK regarding knowledge of cow’s milk allergy also demonstrated significant learning gaps about basic concepts [18]. Although the emphasis of the research was primary prevention of food allergy, rather than diagnosis and management, a Brazilian study of paediatricians, paediatric gastroenterologists, allergists and nutritionists (n = 520), also found gaps in knowledge across all professional groups [19]. In the USA, approximately 60% of primary care and paediatric physicians answered knowledge‒based items correctly in the Chicago Food Allergy Research Survey [20]. However, only 24% were aware that oral food challenges could be used to diagnose food allergy; less than 30% felt confident to interpret biochemical results to diagnose food allergy and only 22% felt their medical training prepared them adequately to care for patients with food allergy. Finally in a South African study of dietitians and medical practitioners [21] (n = 660), 98% of respondents believed they needed more training in food allergy management at undergraduate and postgraduate level.

In our participants, although the majority of respondents used academic journals as a means to maintain CPD and some had attended food allergy conference or courses, the low number of respondents who had completed postgraduate training in food allergy should be emphasised. Further training on food additives and pharmacological reactions was requested by the UK and Australia based respondents, perhaps influenced by the adult dietitians included in both samples. In terms of resources that would be most useful, similar results were seen across the three cohorts, with a handbook, basic course or web-based programme proving most popular.

The use of online training courses has been demonstrated to be effective in increasing postgraduate knowledge in other areas of dietetics such as childhood obesity [22] and infant feeding [23]. Massive Open Online Courses (MOOCs) offer a convenient method to provide distance learning education to dietitians and health professionals internationally, with proven good completion rates and increases in competency [23]. Walsh’s study [18] provides evidence of an improvement in UK doctors’ knowledge of milk allergy using an online training course. Whether this success can be replicated, using a standardized approach across different countries, given the aforementioned differences in undergraduate training, remains to be seen.

There are several limitations to this study. Firstly the response rate of the questionnaires in the UK and Australia was between 3-5%, therefore it is possible that a response bias exists, where those who are interested in food allergy are most likely to participate. Each of the questionnaires was worded slightly differently, in order to adapt the content to local practices (e.g. the questionnaire used in Australia discriminated between FA and FI, the USA and UK based questionnaire did not). The UK questionnaire did not specifically ask the proportion of the caseload composed of allergy patients; instead the question of “how relevant is this questionnaire to your practice” was used as a surrogate to discriminate between those who did and did not work with patients with food allergy. In order to be more inclusive, the UK and Australia questionnaire recruited dietitians who work with both adult and paediatric patients, unlike the original USA based study, which was only aimed at paediatric dietitians. This means the results are not directly comparable. A further limitation is that all the questions were self-rated and therefore subjective. Strengths of the study design are that it included a large number of dietitians (total 797 respondents), with varied years of experience, working in different settings across three different continents.

## Conclusions

There is a need amongst dietitians to increase their knowledge in different aspects of food allergy management, specifically the areas of developing food challenge protocols and management of feeding problems. Dietitians in the UK and Australia identified pharmacological reactions and food additives as the areas of greatest training need and rated a handbook, basic food allergy course or web-based programme as the most preferred methods of learning. Data from these three cohorts provides valuable information for designing food allergy education material for dietitians, which can then be adapted according to country specific needs.

## Electronic supplementary material

Additional file 1: Table S1: Questionnaire used in the United Kingdom. (DOC 57 KB)
