# Supplementary material for: Food allergy competencies of dietitians in the United Kingdom, Australia and United States of America
Source: Clin Transl Allergy. 2014 Nov 14;4:37. doi: 10.1186/2045-7022-4-37 (PMC4405821; doi:10.1186/2045-7022-4-37)
Supplement: Supplementary file 1 — Additional file 1: Table S1: Questionnaire used in the United Kingdom. (DOC 57 KB) [file 13601_2014_1069_MOESM1_ESM.doc]

Additional file 1: Table S1 Questionnaire used in the United Kingdom

| 1 | How long have you worked as a dietitian? |
| --- | --- |
| 2 | What best describes the setting of your practice?  ☐ Hospital (inpatient) ☐ Community ☐ Research ☐ Academic  ☐ Private Practice ☐ Industry ☐ Other |
| 3 | Currently, approximately what percentage of your practice is paediatric patients?  ☐ <10% ☐ 10-24% ☐ 25-49% ☐ 50-74% ☐ 75-99% ☐ 100% |
| 4 | Currently, approximately what percentage of your practice is adult patients?  ☐ <10% ☐ 10-24% ☐ 25-49% ☐ 50-74% ☐ 75-99% ☐ 100% |
| 5 | **Please check the box that indicates your proficiency/knowledge level** |
| 5a | Understanding of the definition of food allergy involving the immune system  ☐ Not at all proficient ☐Low ☐Moderate ☐High ☐ Not applicable in my practice |
| 5b | Understanding of the definition of food intolerances not involving the immune system  ☐ Not at all proficient ☐Low ☐Moderate ☐High  ☐ Not applicable in my practice |
| 5c | Identifying adverse reactions to foods and clinical manifestations (symptoms)  ☐ Not at all proficient ☐Low ☐Moderate ☐High  ☐ Not applicable in my practice |
| 5d | Understanding the steps involved in diagnosing food allergy or intolerance  ☐ Not at all proficient ☐Low ☐Moderate ☐High  ☐ Not applicable in my practice |
| 5e | Creating diagnostic food challenges  ☐ Not at all proficient ☐Low ☐Moderate ☐High  ☐ Not applicable in my practice |
| 5f | Developing/Implementing avoidance diets for children  ☐ Not at all proficient ☐Low ☐Moderate ☐High |
| 5g | Developing/Implementing avoidance diets for adults  ☐ Not at all proficient ☐Low ☐Moderate ☐High  ☐ Not applicable in my practice |
| 5h | Educating patients and families on food avoidance (label reading and cross-contact)  ☐ Not at all proficient ☐Low ☐Moderate ☐High  ☐ Not applicable in my practice |
| 5i | Managing the dietary needs of children with multiple food allergies or intolerances  ☐ Not at all proficient ☐Low ☐Moderate ☐High  ☐ Not applicable in my practice |
| 5j | Managing the dietary needs of adults with multiple food allergies or intolerances  ☐ Not at all proficient ☐Low ☐Moderate ☐High  ☐ Not applicable in my practice |
| 5k | Evaluating safe foods in schools or hospitals  ☐ Not at all proficient ☐Low ☐Moderate ☐High  ☐ Not applicable in my practice |
| 5l | Intervening for childrens’ feeding and behavioural problems caused by food allergies and intolerances  ☐ Not at all proficient ☐Low ☐Moderate ☐High  ☐ Not applicable in my practice |
| 5m | Growth monitoring and deficiencies and how to address this in the allergic/intolerant child  ☐ Not at all proficient ☐Low ☐Moderate ☐High  ☐ Not applicable in my practice |
| 5n | Monitoring nutritional intake and deficiencies and how to address this in the allergic/intolerant adult  ☐ Not at all proficient ☐Low ☐Moderate ☐High  ☐ Not applicable in my practice |
| 6 | Where did you first learn about food allergy/ intolerance?  ☐ Undergraduate training  ☐ BDA paediatric course  ☐ Specialised food allergy conferences  ☐ Postgrad diploma/cert/MSc in allergy  ☐ PhD in allergy  ☐ Self taught/journal article  ☐ Mentored at work  ☐ Continuing education workshop/course e.g. specialist group study day  ☐ No training in food allergies/intolerances  ☐ Other please describe |
| 7 | Which resources would you use to update your knowledge of food allergy/intolerance?  ☐ Professional/Academic publications  ☐ Which resources would you use to update your knowledge:  ☐ Advocacy groups, books, newsletters and articles  ☐ Conference/lecture  ☐ Dietetic textbook  ☐ Academic Web sites  ☐ Other types of websites  ☐ None  ☐ Other |
| 8 | What types of resources do you use to educate your patients with food allergies?  ☐ Diet Sheets  ☐ Audio-visual materials  ☐ Individual teaching sessions  ☐ Group teaching sessions  ☐ Support Groups  ☐ Website  ☐ I do not have any resources  ☐ Other (please specify) |
| 9 | What materials do you use to help your patients follow dietary recommendations?  ☐ Diet sheets on avoidance of specific foods  ☐ Written information on label reading  ☐ Recipes or books  ☐ Shopping information  ☐ Video/DVD  ☐ I do not have any resources  ☐ Other please specify |
| 10 | How likely would you be to use the following material to educate you on FA/FI?  ☐ A handbook on food allergy management with talking points  ☐ Audio visual materials (slide set)  ☐ Web based instructional program  ☐ Access to support groups  ☐ Basic Allergy Course  ☐ Other (please specify) |
| 11 | How likely would you be to use the following material to educate your patients on FA/FI?  ☐ A handbook on food allergy management and education with talking points  ☐ Diet sheets  ☐ Audio visual material  ☐ Web-based information  ☐ Instructional lecture for patients  ☐ Other (please specify) |
| 12 | Which topics are you most in need of further training?  ☐ CMPA ☐ Lactose intolerance ☐ Egg Allergy  ☐ Soya Allergy ☐ Nut and Seed Allergy ☐ Fish/Shellfish Allergy  ☐ Cereal Allergy ☐ Management of IBS ☐ Pharmacology/chemicals  ☐ Oral Allergy Syndrome ☐ Reactions to food additives ☐ Coeliac Disease |
| 13 | How relevant/applicable to your practice were the questions in this survey?  ☐ Not at all ☐ Slightly relevant ☐ Moderately relevant ☐ Very relevant |
